# A randomized controlled trial of a physiology‐guided percutaneous coronary intervention optimization strategy: Rationale and design of the TARGET FFR study

**DOI:** 10.1002/clc.23342

**Published:** 2020-02-10

**Authors:** Damien Collison, John D. McClure, Colin Berry, Keith G. Oldroyd

**Affiliations:** ^1^ West of Scotland Regional Heart and Lung Centre Golden Jubilee National Hospital Clydebank UK; ^2^ Institute of Cardiovascular and Medical Sciences University of Glasgow Glasgow UK

**Keywords:** coronary physiology, fractional flow reserve, non‐hyperemic pressure ratios, PCI optimization, post‐PCI FFR

## Abstract

Post‐percutaneous coronary intervention (PCI) fractional flow reserve (FFR) ≥0.90 confers an improved cardiac prognosis. There are currently limited data available to determine how often it is possible to improve an angiographically acceptable but physiologically suboptimal result. A physiology‐guided optimization strategy can achieve a clinically meaningful increase in the proportion of patients achieving a final post‐PCI FFR ≥0.90 compared to standard care. Following angiographically successful PCI procedures, 260 patients will be randomized (1:1) to receive either a physiology‐guided incremental optimization strategy (intervention group) or blinded post‐PCI coronary physiology measurements (control group). Patients undergoing successful, standard‐of‐care PCI for either stable angina or non‐ST‐segment‐elevation myocardial infarction who meet the study's inclusion and exclusion criteria will be eligible for randomization. The primary endpoint is defined as the proportion of patients with a final post‐PCI FFR result ≥0.90. Secondary endpoints include change from baseline in Seattle Angina Questionnaire and EQ‐5D‐5L scores at 3 months and the rate of target vessel failure and its components (cardiac death, myocardial infarction, stent thrombosis, unplanned rehospitalization with target vessel revascularization) at 3 months and 1 year. 260 individual patients were successfully randomized between March 2018 and November 2019. Key baseline demographics of the study population are reported within. TARGET FFR is an investigator‐initiated, prospective, single‐center, randomized controlled trial of an FFR‐guided PCI optimization strategy. The study has completed recruitment and is now in clinical follow‐up. It is anticipated that primary results will be presented in Autumn 2020. ClinicalTrials.gov Identifier: NCT03259815. [Correction added on Apr 3 2020, after first online publication: Clinical Trials identifier added.]

**Abbreviations**
CFR: coronary flow reserve
dPR: diastolic pressure ratio
FFR: fractional flow reserve
HTG: hyperemic tran‐stent gradient
IC: intracoronary
iFR: instantaneous wave‐free ratio
IMR: index of microcirculatory resistance
ISR: in‐stent restenosis
IVUS: intravascular ultrasound
MACE: major adverse cardiovascular events
NSTEMI: non‐ST segment elevation myocardial infarction
OCT: optical coherence tomography
PCI: percutaneous coronary intervention
Pd/Pa: distal coronary pressure/aortic pressure
PIOS: physiologically guided incremental optimization strategy
RFR: resting full‐cycle ratio
SAQ: Seattle Angina Questionnaire
STEMI: ST‐segment elevation myocardial infarction
TT_hyp_: mean hyperemic transit time
TT_rest_: mean resting transit time

## 1 INTRODUCTION

The utility of fractional flow reserve (FFR) for assessing the physiological significance of coronary stenoses and the benefits of FFR‐guided decision making prior to percutaneous coronary intervention (PCI) have been well‐established in randomized clinical trials.^1^, ^2^ However, FFR is rarely used to assess the final PCI result where standard practice continues to be angiographic assessment alone. From a registry of 750 patients receiving bare metal stents, Pijls et al reported that at 6‐month follow‐up, the lowest event rates occurred in patients with post‐PCI FFR ≥0.90.^3^ Studies and related meta‐analyses involving drug‐eluting stents have suggested similar cutoff values of post‐PCI FFR to predict improved clinical outcomes.^4^, ^5^, ^6^, ^7^, ^8^, ^9^, ^10^, ^11^, ^12^ These range from 0.89 to ≥0.92 with a large systematic review and meta‐analysis of 7470 patients concluding that a post‐PCI FFR ≥0.90 was associated with a significantly lower risk of repeat PCI and major adverse cardiovascular events.^10^ Johnson et al correlated the immediate post‐PCI FFR results from 966 patients with clinical outcomes out to 3 years and demonstrated a significant, inverse relationship between post‐PCI FFR and subsequent clinical events.^13^

Published values for overall mean or median final post‐PCI FFR results range from 0.84 to 0.95.^3^, ^5^, ^6^, ^7^, ^9^, ^12^, ^14^, ^15^, ^16^, ^17^, ^18^, ^19^, ^20^, ^21^, ^22^, ^23^, ^24^, ^25^, ^26^, ^27^, ^28^, ^29^, ^30^, ^31^, ^32^, ^33^, ^34^, ^35^, ^36^, ^37^ The proportion of patients actually achieving a final FFR ≥0.90 varies widely between studies and ranges from 37% to 93%.^3^, ^4^, ^5^, ^6^, ^7^, ^8^, ^16^, ^18^, ^29^, ^38^, ^39^ Of perhaps greater concern however, is the incidence of suboptimal FFR results after stenting. Where reported, the proportion of patients with post‐PCI FFR values ≤0.80 ranges from 4% to 20%.^9^, ^11^, ^24^, ^28^, ^34^, ^37^, ^40^ This indicates that, despite angiographically satisfactory results, as many as one in five patients may have a post‐PCI FFR result that remains below the threshold for performing revascularization in the first place. With up to 38% of patients still reporting angina 1 year after PCI procedures,^41^ it seems plausible that persistently abnormal post‐PCI FFR results may be associated with symptom recurrence.

It has been suggested that non‐hyperemic pressure ratios (NHPRs), such as the instantaneous wave‐free ratio (iFR), also have potential to be used as objective measures of improvement in physiology following PCI.^25^ A recent study employing blinded post‐PCI iFR assessments reported residual ischemia (iFR <0.90) in nearly one in four patients despite angiographically successful stenting results. The authors concluded that 81.6% of these cases were due to inapparent focal lesions potentially amenable to treatment with additional PCI.^42^ While an NHPR‐guided PCI optimization strategy might be more appealing to clinicians as it could facilitate multiple physiological assessments without the need to repeatedly induce hyperemia, data on the prognostic value of post‐PCI NHPR values are currently lacking. The original NHPR, the ratio of distal coronary to aortic pressure (Pd/Pa) is routinely available with all diagnostic guidewires. Recently, two new resting physiology indices have been developed which have diagnostic equivalence to iFR: the diastolic pressure ratio (dPR) and the resting full‐cycle ratio (RFR).^43^, ^44^

One of the reasons clinicians do not routinely measure post‐PCI FFR is that there are currently limited data available to determine how often it is possible to improve an angiographically acceptable but physiologically suboptimal result.

Given the potential prognostic significance of post‐PCI FFR, but general lack of adoption, there is a clear need for randomized controlled trials to:

1. establish the prevalence of suboptimal post‐PCI FFR results in clinical practice;
2. systematically categorize the remediable mechanisms where this occurs;
3. establish which PCI optimization strategies can effectively increase the proportion of patients with functionally optimal revascularization.

## 2 METHODS

**Figure 1:**
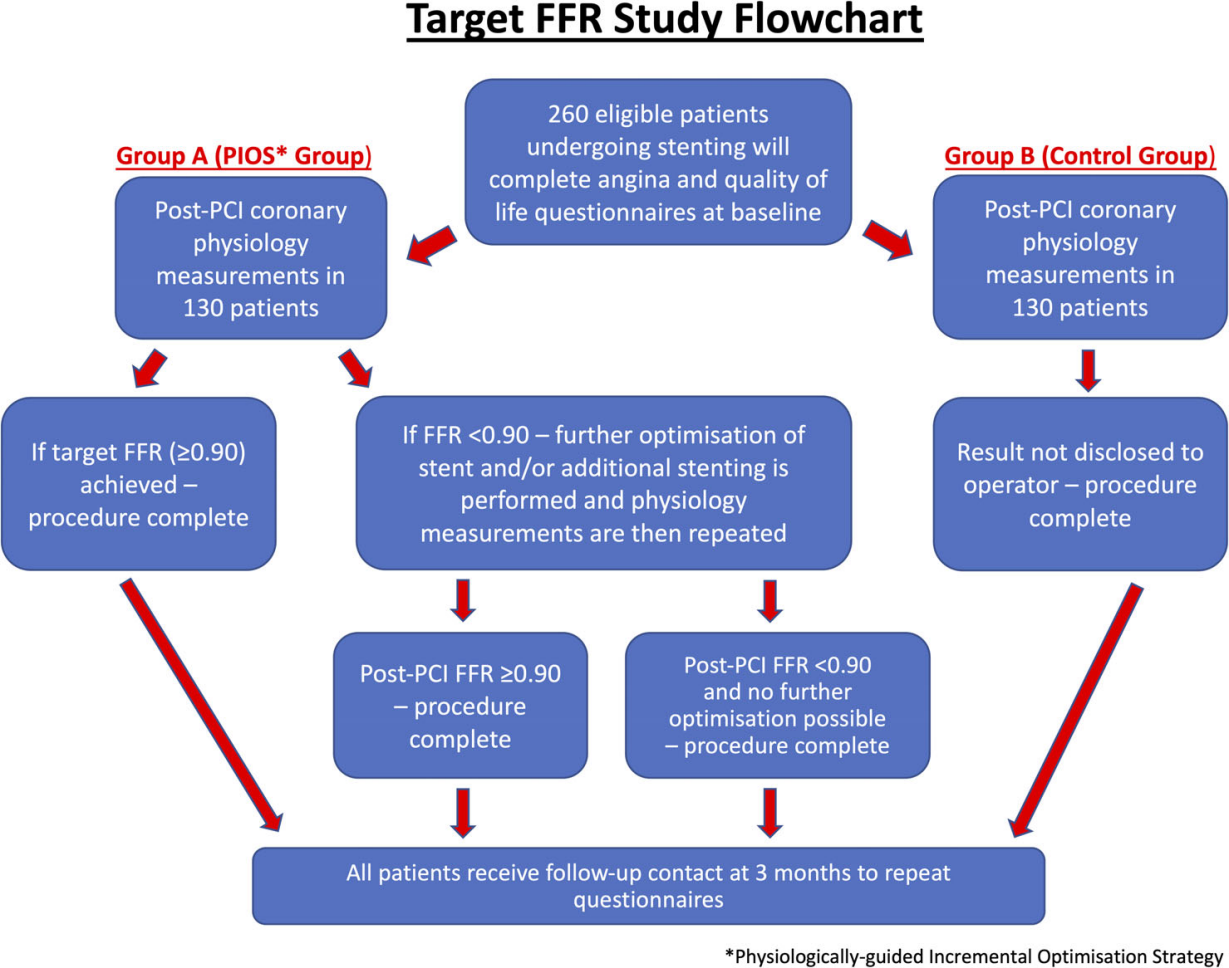
Target FFR study flowchart. PIOS—physiologically guided incremental optimization strategy

TARGET FFR is an investigator‐initiated, prospective, single‐center, randomized controlled trial of an FFR‐guided PCI optimization strategy. The primary hypothesis is that this strategy will result in a clinically meaningful increase in the proportion of patients achieving a final post‐PCI FFR ≥0.90 compared to standard care. Following angiographically successful PCI procedures, 260 patients will be randomized (1:1) to receive either a physiology‐guided incremental optimization strategy (PIOS intervention group) or blinded post‐PCI coronary physiology measurements (control group). Patients undergoing successful, standard‐of‐care PCI for either stable angina or Non‐ST segment elevation myocardial infarction (NSTEMI) who meet the study's inclusion and exclusion criteria (Table 1) will be eligible for randomization. All patients will complete the Seattle Angina Questionnaire (SAQ‐7) and EQ‐5D‐5L questionnaire both prior to, and 3 months after, their procedure. Longer term outcomes will be assessed using record linkage. The study flowchart is depicted in Figure 1.

**Table 1:** Inclusion/exclusion criteria

Inclusion criteria Patients >18 years of age with coronary artery disease including stable angina and NSTEMI Participants must be able to provide informed consent
Exclusion criteria PCI in a coronary artery bypass graft PCI to an ISR lesion PCI to a target artery providing Rentrop grade 2 or 3 collateral blood supply to another vessel Inability to receive adenosine (eg, severe reactive airway disease, marked hypotension, or advanced atrioventricular block without pacemaker). Recent (within 1 week prior to cardiac catheterisation) STEMI in any arterial distribution (not specifically target lesion). Severe cardiomyopathy (LVEF <30%). Renal insufficiency such that an additional 20 to 30 mL of contrast would, in the opinion of the operator, pose unwarranted risk to the patient.

Abbreviations: ISR, in‐stent restenosis; LVEF—left ventricular ejection fraction; NSTEMI, non‐ST segment myocardial infarction; PCI, percutaneous coronary intervention; STEMI—ST‐segment elevation myocardial infarction.

### 2.1 Study endpoints

The primary endpoint is defined as the proportion of patients with a final post‐PCI FFR result ≥0.90.

Secondary endpoints include:

1. the proportion of patients with FFR ≤0.80;
2. the change from baseline in SAQ‐7 at 3 months;
3. the change from baseline in EQ‐5D‐5L scores at 3 months;
4. the rate of target vessel failure (TVF) and its components (cardiac death, myocardial infarction, stent thrombosis, unplanned rehospitalization with target vessel revascularization) at 3 months and 1 year.

A complete list of secondary outcome measures is provided in Table 2.

**Table 2:** Secondary outcome measures of the target FFR study

*Patient‐reported outcome measures* Change from baseline in SAQ and EQ‐5D‐5L scores at 3 months
*3‐month and 1‐year follow‐up* The rate of TVF and its components (cardiac death, myocardial infarction, stent thrombosis, unplanned rehospitalization with target vessel revascularization)
*Additional coronary physiology outcomes* The proportion of patients with final post‐PCI FFR ≤0.80 The proportion of patients with final post‐PCI dPR ≥0.90 The proportion of patients with final post‐PCI RFR ≥0.90 The proportion of patients with final post‐PCI CFR ≥2.0 The proportion of patients with final post‐PCI IMR >25 The proportion of patients with final post‐PCI IMRc >25 ΔFFR from pre‐PCI to final post‐PCI value ΔdPR from pre‐PCI to final post‐PCI value ΔRFR from pre‐PCI to final post‐PCI value ΔCFR from pre‐PCI to final post‐PCI value Δ TT_rest_ from pre‐PCI to final post‐PCI value Δ TT_hyp_ from pre‐PCI to final post‐PCI value ΔIMR from pre‐PCI to final post‐PCI value ΔIMRc from pre‐PCI to final post‐PCI value ΔFFR from pre‐PCI to final post‐PCI value Percent FFR change from pre‐PCI to final post‐PCI value Percent dPR change from pre‐PCI to final post‐PCI value Percent RFR change from pre‐PCI to final post‐PCI value Percent CFR change from pre‐PCI to final post‐PCI value Percent TT_rest_ change from pre‐PCI to final post‐PCI value Percent TT_hyp_ change from pre‐PCI to final post‐PCI value Percent IMR change from pre‐PCI to final post‐PCI value Percent IMRc change from pre‐PCI to final post‐PCI value
*Procedural characteristics* Procedure duration Cost of additional equipment employed in the experimental arm Fluoroscopy dose Contrast material dose Incidence of procedural complications such as coronary artery dissection or perforation. Incidence of significant pressure wire drift (≥ ±0.04)
*Additional analyses* An “as‐treated” analysis of the primary and preceding secondary outcome measures

Abbreviations: CFR, coronary flow reserve; dPR, diastolic pressure ratio; FFR, fractional flow reserve; IMR, index of microcirculatory resistance; IMRc, corrected index of microcirculatory resistance; RFR, resting full‐cycle ratio; SAQ, Seattle Angina Questionnaire; TT_hyp_, mean hyperemic transit time; TT_rest_, mean resting transit time; TVF, target vessel failure.

### 2.2 Study procedures

PCI procedures will be performed according to standard clinical practice. Treatment decisions (including the use of adjunctive intracoronary imaging such as OCT or IVUS) and the definition of an angiographically acceptable PCI result will be left entirely to the operator's judgment. The study protocol mandates that all enrolled patients should have pre‐PCI coronary physiology assessments performed, however, the decision whether to then use the pressure wire for PCI or employ an alternative angioplasty wire is at the operator's discretion. Decisions on choice and duration of antiplatelet medications and/or combination with anticoagulant therapy will also be left to the operator's clinical judgment. Patients will only be randomized and post‐PCI study measurements/interventions performed after the operator has declared the standard care procedure to be complete.

### 2.3 PIOS intervention group (group A)

If post‐PCI FFR is <0.90, the coronary physiology results and hyperemic pullback assessment will be disclosed to the operator. Based on the interpretation of the pullback recording, the operator will attempt to obtain the target optimal post‐PCI FFR result by following the steps of the PIOS algorithm outlined below:

1. If the residual pressure gradient is interpreted to reflect diffuse atherosclerosis with no focal step‐ups, the result is accepted and no optimization is attempted.
2. If there is a hyperemic trans‐stent gradient (HTG) ≥0.05 further postdilation with a larger non‐compliant balloon to at least 18 atm should be performed followed by repeat FFR. Additionally, the operator may choose to employ intracoronary imaging (IVUS or OCT) to guide postdilation/optimization of the stented segment.
3. If there is a step‐up of ≥0.05 across a relatively focal (<20 mm) unstented segment that is technically suitable for further stenting, then a further stent should be deployed followed by repeat FFR.
4. If the FFR remains <0.90 after steps B ± C, a further FFR pullback will be performed. If the criteria for further stent optimization or implantation are again met, additional postdilation should be undertaken and/or one additional stent may be deployed followed by a final FFR pullback.

### 2.4 Control group (group B)

In keeping with standard care, the operator will determine procedural completeness and success based on the angiographic and/or intracoronary imaging appearances. Post‐PCI coronary physiology measurements will be performed but not disclosed to the operator. No further intervention or optimization measures will be undertaken.

### 2.5 Follow‐up

Follow‐up will be performed by clinical research nurses blinded to both the assigned treatment arm and the final FFR result. All patients will be contacted by letter and/or telephone 3 months after their PCI procedure to repeat the SAQ‐7 and EQ‐5D‐5L questionnaires. In cases where an adverse event or clinical endpoint has occurred, additional information will be retrieved from the patient's electronic health record or general practitioner. Clinical outcomes will be reviewed again at 1 year using electronic health record linkage.

### 2.6 Pilot data, power, and sample size calculation

Pilot data from 50 patients who underwent post‐PCI FFR assessment at our institution revealed that an initial post‐PCI FFR result ≥0.90 was achieved in only 16/50 (32%). Additional optimization measures (further postdilation, stenting or both) were attempted in just nine patients (18%). Initial post‐PCI FFR increased from a median of 0.83 (0.80‐0.86) to a final FFR of 0.88 (0.86‐0.89) in these patients. The results of pullback measurements performed in all 50 vessels, however, revealed at least one target for additional optimization measures was present in up to 40% of patients (HTG ≥0.05 in 19/50 (38%); focal step‐up ≥0.05 either proximal or distal to the stented segment in 21/50 (42%). It is hypothesized that systematically applying the PIOS intervention will result in a 20% absolute increase in the proportion of patients with a final post‐PCI FFR ≥0.90 compared to the control group. A sample size of 130 patients per group would be required to have 90% power to detect a 20% difference between groups at the 5% significance level; therefore, 260 patients will be randomized. Patients presenting with stable angina or NSTEMI who attend our institution for diagnostic coronary angiography proceed to PCI during the same procedure in approximately 40% of cases. It is therefore estimated that approximately 650 patients will be enrolled in the study in order to randomize 260 patients following their standard‐of‐care PCI.

### 2.7 Statistical analysis

The study data will be summarized for the randomized population overall, and by randomized group. The number of observations and number of missing values will be reported. Continuous variables will have normality tests applied and be summarized using the mean ± SD or median and interquartile range according to their distribution. Differences in continuous variables between randomized groups will be assessed using Student's *t* tests or Mann‐Whitney *U* tests as appropriate. The Pearson correlation coefficient will be applied to parametric variables while correlation between nonparametric variables will be assessed using Spearman's rank correlation. Categorical variables will be summarized with frequencies and percentages. Differences in categorical variables between randomized groups will be evaluated using Chi‐square tests or Fisher's exact tests.

Where relevant, changes from baseline will be summarized. Multivariate logistic regression analyses will be employed to assess for clinical predictors of post‐PCI FFR values ≥0.90 and ≤0.80. The primary outcome will be summarized in the full analysis set as a whole and by treatment group. A test and 95% CI for two proportions (adjusted Wald method) will be employed, together with Fisher's exact test. Additional secondary analyses on this outcome will use logistic regression to investigate whether any of the baseline characteristics affect the outcome. This will be performed by first investigating each characteristic on its own (together with the treatment group). Any variables that are significant here will be added to build a larger model, bearing in mind sample size limitations. For the binary categorical secondary outcomes, the same analysis approach will be used as with the primary outcome. For quantitative secondary outcomes, two sample *t* tests or Mann Whitney *U* tests will be used as appropriate, as well as further analyses using regression to investigate whether any of the baseline characteristics affect the outcome. All tests will be two sided and a *P*‐value of <.05 will be considered significant. Efficacy analyses will be carried out according to the intention to treat principle, that is, in relation to randomized treatment allocation, rather than treatment received. Safety analyses will be carried out in relation to treatment received. Details of subgroup and additional analyses are provided in the Supplementary Appendix.

### 2.8 Study organization

The study received ethical approval from the West of Scotland Research Ethics Service (17/WS/0153) and will be conducted in accordance with the International Conference on Harmonization Good Clinical Practice (ICH GCP) Guideline and the Declaration of Helsinki (64th World Medical Association General Assembly, Fortaleza, Brazil, October 2013). The study sponsor is the NHS National Waiting Times Centre Board (Golden Jubilee National Hospital). Details of the Trial Steering Committee, Clinical Endpoints Committee, and Physiology Core Lab are provided in the Supplementary Appendix.

## 3 RESULTS

Between March 8, 2018 and November 22, 2019, we successfully randomized 260 patients meeting the trial's inclusion and exclusion criteria. Preliminary baseline characteristics of the study population are presented in Table 3. The trial is now in clinical follow‐up and it is anticipated the primary results will be presented in Autumn 2020.

**Table 3:** Baseline patient demographics (preliminary results)

	Total (*n* = 260)	PIOS (*n* = 131)	Control (*n* = 129)
Male	226 (86.9%)	117 (89.3%)	109 (84.5%)
Age	59 (54‐66)	58 (54‐66)	60 (55‐68)
BMI	29 (27‐32)	29 (26‐32)	29 (27‐32)
Hypertension	116 (44.6%)	58 (44.3%)	58 (45%)
Hypercholesterolemia	146 (56.2%)	72 (55%)	74 (57.4%)
Diabetes	49 (18.8%)	24 (18.3%)	25 (19.4%)
OHAs	42 (85.7%)	21 (87.5%)	21 (84%)
Insulin	5 (10.2%)	3 (12.5%)	2 (8%)
Atrial fibrillation	19 (7.3%)	10 (7.6%)	9 (7%)
OAC	13 (68.4%)	6 (60%)	7 (77.8%)
CHA2DS2‐Vasc			
2	6 (31.6%)	3 (15.8%)	3 (15.8%)
3	4 (21.1%)	3 (30%)	1 (11.1%)
4	4 (21.1%)	2 (20%)	2 (22.2%)
5	4 (21.1%)	2 (20%)	2 (22.2%)
6	1 (5.3%)	0	1 (11.1%)
Previous TIA/stroke	17 (6.5%)	8 (6.1%)	9 (7%)
CKD^a^	5 (1.9%)	3 (2.3%)	2 (1.6%)
Family history of CAD	172 (66.2%)	88 (67.2%)	84 (65.1%)
History of smoking	183 (70.4%)	92 (70.2%)	91 (70.5%)
Current	50 (27.3%)	28 (30.4%)	22 (24.2%)
Within past year	41 (22.4%)	22 (23.9%)	19 (20.9%)
Ex‐smoker >1 y	92 (50.3%)	42 (45.7%)	50 (54.9%)
Thyroid dysfunction	20 (7.7%)	9 (6.9%)	11 (8.5%)
Heart failure	44 (16.9%)	28 (21.4%)	16 (12.4%)
NYHA class 1	29 (65.9%)	19 (67.9%)	10 (62.5%)
NYHA class 2	15 (34.1%)	9 (32.1%)	6 (37.5%)
HFrEF	43 (97.7%)	28 (100%)	15 (93.8%)
Previous MI	95 (36.5%)	50 (38.2%)	45 (34.9%)
Previous PCI	100 (38.5%)	54 (41.2%)	46 (35.7%)
Previous CABG	1 (0.4%)	1 (0.8%)	0
Valvular heart disease	8 (3.1%)	2 (1.5%)	6 (4.7%)
Aortic stenosis	6 (2.3%)	1 (0.8%)	5 (3.9%)
Mitral regurgitation	2 (0.8%)	1 (0.8%)	1 (0.8%)
Angina	215 (82.7%)	107 (81.7%)	108 (83.7%)
CCS class 1	58 (27%)	28 (26.2%)	30 (27.8%)
CCS class 2	101 (47%)	51 (47.7%)	50 (46.3%)
CCS class 3	55 (25.6%)	27 (25.2%)	28 (25.9%)
CCS class 4	1 (0.5%)	1 (0.9%)	0
Cardiac medications			
Single APT	253 (97.3%)	128 (97.7%)	125 (96.9%)
Dual APT	185 (71.2%)	97 (74.1%)	88 (68.2%)
OAC	16 (6.2%)	8 (6.1%)	8 (6.2%)
Statin	250 (96.2%)	127 (97%)	123 (95.4%)
Beta blocker	237 (91.2%)	121 (92.4%)	116 (89.9%)
CCB	52 (20%)	22 (16.8%)	30 (23.3%)
ACEI	175 (67.3%)	91 (69.5%)	84 (65.1%)
ARB	23 (8.9%)	11 (8.4%)	12 (9.3%)
Diuretic	30 (11.5%)	13 (9.9%)	17 (13.2%)
GTN spray	123 (47.3%)	61(46.6%)	62 (48.1%)
Used daily	30 (24.4%)	13 (21.3%)	17 (27.4%)
Used weekly	67 (54.55)	34 (55.7%)	32 (51.6%)
Used monthly	27 (22%)	14 (23%)	13 (21%)
Oral nitrate	69 (26.5%)	26 (19.9%)	43 (33.3%)
Nicorandil	22 (8.5%)	14 (10.7%)	8 (6.2%)
Ivabradine	5 (1.9%)	3 (2.3%)	2 (1.6%)
No. anti‐anginal meds			
0	9 (3.5%)	4 (3.1%)	5 (3.9%)
1	99 (38.1%)	55 (42%)	44 (34.1%)
2	114 (43.8%)	55 (42%)	59 (45.7%)
3	31 (11.9%)	13 (9.9%)	18 (14%)
4	7 (2.7%)	4 (3.1%)	3 (2.3%)
Indication			
Stable angina	88 (33.9%)	40 (30.5%)	48 (37.2%)
Staged PCI	16 (18.2%)	8 (20%)	8 (16.7%)
ACS‐NSTEMI	101 (38.8%)	50 (38.2%)	51 (39.5%)
Days post‐MI	21 (12‐28.5)	20 (7‐26.3)	23 (16‐31)
ACS‐unstable angina	3 (1.2%)	2 (1.5%)	1 (0.8%)
Staged PCI/completion of revascularization	68 (26.2%)	39 (29.8%)	29 (22.5%)
Post‐STEMI	46 (67.7%)	29 (74.4%)	17 (58.6%)
Days since MI	68.8±29.5	70.4±30.9	66.1±27.6
Post‐NSTEMI	22 (32.4%)	10 (25.6%)	12 (41.4%)
Days since MI	67 (54‐98)	64 (54‐86.8)	79.5 (53.3‐110.8)
Target vessel			
LAD	149 (57.3%)	75 (57.3%)	74 (57.4%)
RCA	67 (25.8%)	28 (21.4%)	39 (30.2%)
LCx	33 (12.7%)	20 (15.3%)	13 (10.1%)
OM	10 (3.8%)	8 (6%)	2 (1.6%)
Diagonal	1 (0.4%)	0	1 (0.8%)

a All five patients had stage 3a CKD (eGFR 45‐59): mild‐moderate renal impairment.

Abbreviations: ACEI, angiotensin converting enzyme inhibitor; ACS, acute coronary syndrome; APT, antiplatelet therapy; ARB, angiotensin II‐receptor blocker; BMI, body mass index; CABG, coronary artery bypass grafting; CAD, coronary artery disease; CCB, calcium channel blocker; CCS, Canadian cardiovascular society; CKD, chronic kidney disease; eGFR, estimated glomerular filtration rate; GTN, glyceryl trinitrate; HFrEF, heart failure with reduced ejection fraction; LAD, left anterior descending; LCx, left circumflex; MI, myocardial infarction; NSTEMI, non‐ST‐segment elevation myocardial infarction; OAC, oral anticoagulant; OHAs, oral hypoglycemic agents; OM, obtuse marginal; PCI, percutaneous coronary intervention; RCA, right coronary artery; STEMI, ST‐segment elevation myocardial infarction.

## 4 DISCUSSION

Prior studies of post‐PCI FFR have recruited patients with either stable angina or recent NSTEMI. However, while in general enrolling heterogeneous populations enhances external validity, in this case, the performance of the diagnostic test may differ according to the nature of the coronary artery disease. By measuring CFR and IMR with FFR we aim to take account of microvascular dysfunction to help inform the interpretation of the primary and secondary physiology outcomes. The sample size calculation is based on pilot data which identified potential targets for additional intervention following an index PCI procedure. The indication for the PIOS intervention may be more limited if a high proportion of patients have a post‐PCI FFR ≥0.90 or if there is a higher incidence of the diffuse gradient disease pattern in the population than predicted. This could result in the study being underpowered. Currently, there are limited data on the potential to further optimize the physiological result of a PCI procedure. Agarwal et al reported that 137 of the 664 lesions (20.6%) in their patient cohort underwent additional PCI based on the presence of a persistently ischemic, or if not ischemic, “unsatisfactory” (as determined by the operator) initial post‐PCI FFR.^7^ The mean initial FFR for these lesions was 0.78 ± 0.07. 58/137 lesions (42%) received further postdilation of the implanted stent with a bigger balloon size and higher pressure and duration of inflation. 45/137 (33%) had another stent implanted while 24/137 (18%) underwent both additional stenting and balloon postdilation. These subsequent interventions led to an improvement in FFR in this subgroup from 0.78 ± 0.07 to 0.87 ± 0.05. Overall, suboptimal initial post‐PCI FFR prompting subsequent intervention led to an increase in lesions with final FFR >0.91 from 34% to 43% (≥0.86 from 60% to 74%) and decreased persistently ischemic lesions (≤0.81) from 21% to 9%. Of note, however, only 74/118 (63%) of those with initial post‐PCI FFR ≤0.81 actually had further intervention attempted. In a cohort of 13 patients who fulfilled both functional and OCT‐defined criteria for suboptimal stent results, Wolfrum et al increased the mean post‐PCI FFR in this group from 0.80 ± 0.02 to 0.88 ± 0.01 through a combination of additional stent postdilation (46%), additional stenting (39%) or a combination of both strategies (15%). Larger increases in FFR value were observed in the seven patients who received additional stents.^45^

The proposed target for an “optimal” post‐PCI FFR result stems from a meta‐analysis which included several early studies that assessed post‐PCI FFR using relatively small bolus doses of intracoronary adenosine to induce hyperemia. Smaller doses of adenosine may not have achieved maximal hyperemia and could potentially have overestimated the final FFR results. As such, a post‐PCI FFR value of ≥0.90 may not be realistic target, particularly in the left anterior descending artery which typically subtends a larger myocardial mass. It has in fact been suggested that optimal cutoff values of post‐PCI FFR are different according to the target vessels involved.^46^ Consequently, the PIOS intervention may not be effective at increasing the FFR value to such an extent, or at least, not in the proportions desired.

### 4.1 Limitations

There are potential concerns regarding both performance and confirmation bias with this trial design and as such the following steps were taken to minimize their effects.

By its very nature, the PIOS group potentially receives more focused attention from the interventionalist than the control group. Despite the operator's best efforts, this does not necessarily translate into higher final FFR values (the primary endpoint), however, and that is the question the trial seeks to answer—does routinely applying a post‐PCI physiology‐guided optimization strategy actually achieve a clinically meaningful difference in the proportion of patients with optimal final FFR results?

Following PCI procedures, the treating interventionalists reassured all patients that they received the highest standard of care, regardless of their randomization group. Specific results of the final physiology measurements were not disclosed to patients in either group.

In an effort to mitigate the potential Hawthorne (“observer”) effect, the study could have on local PCI practices, other than cases randomized to the PIOS intervention where post‐PCI FFR was <0.90, operators were blinded to all post‐PCI physiology results, 15 different interventional cardiologists were enlisted to participate in the trial in order to: (a) assess a wider variety of practice and (b) dilute the exposure any one physician had to unblinded post‐PCI physiology results (on average each operator would have performed less than 10 unblinded PIOS cases over a 20‐month period). Further details on blinding procedures are contained in the Supplementary Appendix.

Furthermore, we posit that rather than being subject to confirmation bias, the primary results of the trial will actually challenge such bias regarding the functional results of PCI procedures.

## 5 SUMMARY

TARGET FFR is an investigator‐initiated, prospective, single‐center, randomized controlled trial to determine the feasibility and efficacy of using a coronary physiology‐guided optimization strategy to achieve final post‐PCI FFR results ≥0.90.

## CONFLICT OF INTERESTS

Damien Collison has received speaker fees from Abbott. Colin Berry is employed by the University of Glasgow which holds consultancy and research agreements with Abbott, AstraZeneca, Boehringer Ingelheim, GSK, HeartFlow, Menarini, Novartis, Ospens, and Siemens Healthcare. Keith G. Oldroyd has received consultant and speaker fees from Abbott and Boston Scientific. John D. McClure has no conflicts of interest to disclose.

## Supporting information


**Appendix** **S1.** Supporting Information.
