# Supplementary material for: A randomized controlled trial of a physiology‐guided percutaneous coronary intervention optimization strategy: Rationale and design of the TARGET FFR study
Source: Clin Cardiol. 2020 Feb 10;43(5):414–22. doi: 10.1002/clc.23342 (PMC7244297; doi:10.1002/clc.23342)
Supplement: Supplementary file 1 — Appendix S1. Supporting Information. [file CLC-43-414-s001.docx]

**Supplementary Appendix**

Table of Contents

1. Trial Organisation and Committees
2. Randomisation and Blinding
3. Coronary Physiology Measurements
4. Coronary Physiology Core Laboratories
5. Clinical Endpoint Definitions
6. Subgroup Analyses
7. Additional Analyses
8. References
9. *Trial Organisation and Committees*

*Principal Investigator:*

Professor Keith G. Oldroyd, Golden Jubilee National Hospital, Glasgow, UK

*Steering Committee:*

Professor Keith G. Oldroyd (chair), Golden Jubilee National Hospital, Glasgow, UK

Dr. Damien Collison, Golden Jubilee National Hospital and University of Glasgow, Glasgow, UK

Professor Colin Berry, Golden Jubilee National Hospital and University of Glasgow, Glasgow, UK

*Clinical Endpoints Committee:*

Professor Mark Petrie (chair), Glasgow Royal Infirmary and University of Glasgow, Glasgow, UK

Dr. Ross Campbell, Queen Elizabeth University Hospital, Glasgow, UK

Dr. Barry Hennigan, Mater Private Cork, Cork, Republic of Ireland

1. *Randomisation and Blinding*

Randomisation and electronic Case Report Form (eCRF) services will be provided through a secure (ISO 27001 & 9001 compliant) web-based platform which is compliant with GCP regulations, Annex 11 and 21 CFR Part 11 (Castor EDC, Amsterdam, Netherlands). Randomisation will be performed using a 1:1 variable block (2,4,6) randomization method generated from within the study’s eCRF platform. Patients will not be informed of their final coronary physiology results. FFR results will not be displayed on the procedure-room monitor and the physiology interface will only be visible to research staff in the control room. The FFR results will only be disclosed to the operator for patients with initial post-PCI FFR <0.90 who are randomised to receive the optimisation strategy. The rationale for maintaining operator-blinding for all other post-PCI physiology results is to try and minimise the potential Hawthorne effect (‘observer effect’) the study could have on local PCI practices.

1. *Coronary Physiology Measurements*

Coronary physiology measurements will be performed using the PressureWire X Guidewire (Abbott Laboratories, Illinois, U.S.A.) and analysed in real time using dedicated software (CoroFlow v3.0, Coroventis Research AB, Uppsala, Sweden). The standard operating procedure for the Target FFR coronary physiology assessment is outlined below.

Following administration of a 200mcg bolus of intracoronary nitrate to the study artery, the pressure wire will be advanced to the tip of the guide catheter and equalized with the aortic pressure. The pressure wire will then be advanced to the distal vessel. Pd/Pa is the ratio of resting aortic to distal coronary artery blood pressure. FFR is defined as the mean distal coronary artery pressure divided by mean aortic pressure during maximal hyperaemia. Hyperaemia will be induced by infusion of adenosine into an antecubital vein at a rate of 140mcg/kg/min. In addition to resting Pd/Pa, the following non-hyperaemic pressure ratios will also be measured:

- dPR (the Pd/Pa ratio of the averaged Pa and Pd values measured during the entire diastolic periods of 5 consecutive cardiac cycles)
- RFR (the lowest Pd/Pa ratio over an entire cardiac cycle averaged over 5 consecutive cardiac cycles)

Using a thermodilution technique, the mean transit times of three 3ml intracoronary boluses of room-temperature saline will be recorded at rest and again during hyperaemia. This will allow Coronary Flow Reserve (CFR - the ratio of resting to hyperaemic coronary flow) and the Index of Microcirculatory Resistance (IMR - the product of mean hyperaemic distal coronary pressure and mean hyperaemic transit time) to be calculated as previously described(1-4). FFR will be measured during stable hyperaemia with the pressure wire sensor (located 30mm proximal to the wire tip) positioned as far distally in the vessel as practical - the operator will be encouraged to match the position a standard angioplasty wire would usually occupy in the artery. This will allow for a more accurate approximation of the myocardial FFR value which can be overestimated with more proximal sensor positions. Finally, a hyperaemic pressure wire pullback assessment will be performed and the sensor will be returned to the tip of the guide catheter to assess for pressure drift. If there is a drift value of >0.03, the measurements should be repeated a second time. Using the CoroFlow software, research staff will annotate the hyperaemic pullback recording to co-register the anatomical landmarks passed during fluoroscopy-guided pullback of the pressure wire (distal and proximal stent edges, the position of relevant side branches and the tip of the guiding catheter etc.). This will allow calculation of the hyperaemic trans-stent gradient (HTG) and any residual pressure gradients proximal or distal to the stented segment.

1. Coronary Physiology Core Laboratories

Due to nature of the trial, clinical decisions pertaining to revascularisation and optimisation measures will be undertaken in real-time on the basis of the on-site coronary physiology measurements.

For validation purposes, post-hoc core lab analysis will be performed by Coroventis Research Ab, Uppsala, Sweden.

Additional core lab analysis of Fractional Flow Reserve (FFR) values (using the Smart Minimum FFR algorithm) will be undertaken by Professor Nils Johnson, University of Texas, USA.

Additional core lab analysis of Diastolic Pressure Ratio (dPR) will be undertaken by Dr. Marcel van’t Veer, Catharina Hospital, Eindhoven, The Netherlands.

Site and core-lab values will be reported.

1. Clinical Endpoint Definitions

Table S1. Clinical Endpoint Definitions(5)

| Endpoint | Definition |
| --- | --- |
| Death | The cause of death will be adjudicated as being due to cardiovascular causes,  non-cardiovascular causes, or undetermined causes.  • Cardiovascular death includes sudden cardiac death, death due to acute myocardial infarction (MI), heart failure or cardiogenic shock, stroke, other cardiovascular causes, or bleeding  • Non-cardiovascular death is defined as any death with known cause not of  cardiac or vascular causes  • Undetermined cause of death refers to a death not attributable to one of the  above categories of cardiovascular death or to a non-cardiovascular cause.  For this trial all deaths of undetermined cause will be included in the  cardiovascular category |
| Myocardial Infarction | In this trial myocardial infarction will be defined according to the Fourth Universal Definition of Myocardial Infarction (2018)(6) |
| Stroke | The rapid onset of a new persistent neurologic deficit attributed to an obstruction in cerebral blood flow and/or cerebral haemorrhage with no apparent non-vascular cause (e.g., trauma, tumour, or infection). Available neuroimaging studies will be considered to support the clinical impression and to determine if there is a demonstrable lesion compatible with an acute stroke.  Strokes will be classified as ischemic, haemorrhagic, or unknown.  Four criteria must be fulfilled to diagnosis stroke:  1. Rapid onset of a focal/global neurological deficit with at least one of the following: change in level of consciousness, hemiplegia, hemiparesis, numbness or sensory loss affecting one side of the body, dysphasia/aphasia, hemianopia, amaurosis fugax, other new neurological sign(s)/symptom(s) consistent with stroke; and  2. Duration of a focal/global neurological deficit ≥24 hours or <24 hours if any of the following conditions exist:  i. At least one of the following therapeutic interventions:  a. Pharmacologic (i.e., thrombolytic drug administration)  b. Non-pharmacologic (i.e., neuro-interventional procedure such as intracranial angioplasty)  ii. Available brain imaging clearly documents a new haemorrhage or infarct  iii. The neurological deficit results in death  3. No other readily identifiable non-stroke cause for the clinical presentation (e.g., brain tumour, trauma, infection, hypoglycaemia, other metabolic abnormality, peripheral lesion, or drug side effect). Patients with non-focal global encephalopathy will not be reported as a stroke without unequivocal evidence based upon neuroimaging studies.  4. Confirmation of the diagnosis by a specialist and at least one of the following:  a. Brain imaging procedure (at least one of the following):  i. CT scan  ii. MRI scan  iii. Cerebral vessel angiography  b. Lumbar puncture (i.e. spinal fluid analysis diagnostic of intracranial haemorrhage) |
| Target Vessel Revascularisation | The target vessel is defined as the entire major coronary vessel proximal and distal to the target lesion including upstream and downstream branches and the target lesion itself. Target vessel revascularization is defined as any repeat percutaneous intervention or surgical bypass of any segment of the target vessel including the target lesion.  Revascularisation will be considered ischaemia-driven if the diameter stenosis of the revascularised coronary segment is ≥50% by Quantitative Coronary Angiography (QCA) and any of the following criteria for ischemia are met:  • A positive functional study corresponding to the area served by the target lesion; or  • Ischaemic ECG changes at rest in a distribution consistent with the target vessel; or  • Typical ischemic symptoms referable to the target lesion; or  • IVUS of the target lesion with a minimal lumen area (MLA) of ≤4 mm2 for non-left main lesions or ≤6 mm2 for left main lesions.  If the lesions are de novo (i.e. not restenotic), the plaque burden must also be ≥60%; or  • FFR of the target lesion ≤0.80  A target lesion revascularisation for a diameter stenosis <50% might also be considered ischaemia-driven by the Clinical Events Committee if there was a markedly positive functional study or ECG changes corresponding to the area served by the target lesion. |
| Stent Thrombosis | *Definite Stent Thrombosis*  Angiographic confirmation of stent thrombosis  The presence of a thrombus^†^ that originates in the stent or in the segment 5 mm proximal or distal to the stent or in a side branch originating from the stented segment and the presence of at least 1 of the following criteria:   - Acute onset of ischemic symptoms at rest - New electrocardiographic changes suggestive of acute ischaemia - Typical rise and fall in cardiac biomarkers (refer to definition of spontaneous myocardial infarction)    Or  Pathological confirmation of stent thrombosis   - Evidence of recent thrombus within the stent determined at autopsy - Examination of tissue retrieved following thrombectomy (visual/histology)   *Probable Stent Thrombosis*  Regardless of the time after the index procedure, any myocardial infarction that is related to documented acute ischaemia in the territory of the implanted stent without angiographic confirmation of stent thrombosis and in the absence of any other obvious cause.^‡^  *Silent Stent Thrombosis*  The incidental angiographic documentation of stent occlusion in the absence of clinical signs or symptoms is not considered stent thrombosis.  *Timing of Stent Thrombosis (duration after stent implantation)*   Acute : 0^§^–24 hours   Subacute: >24 hours–30 days   Late: 30 days–1 year   Very late: >1 year  Early stent thrombosis is 0 to 30 days (acute plus subacute stent thrombosis).  †Occlusive thrombus: Thrombolysis in Myocardial Infarction (TIMI) grade 0 or 1 flow within or proximal to a stent segment. Non-occlusive thrombus: intracoronary thrombus is defined as a (spherical, ovoid, or irregular) non-calcified filling defect or lucency surrounded by contrast material (on 3 sides or within a coronary stenosis) seen in multiple projections, persistence of contrast material within the lumen, or visible embolisation of intraluminal material downstream.  ‡When the stented segment is in the left circumflex coronary artery or in the presence of pre-existing electrocardiographic abnormalities (eg, left bundle branch block, paced rhythms), definitive evidence of localization may be absent and Clinical Events Committee adjudication is based on review of all available evidence).  §Defined as the moment the patient is undraped and taken off the catheterisation table. |

1. *Subgroup Analyses*

The following subgroup variables will be considered:

• Age

• Sex

• Acute Coronary Syndrome

• Smoking Status

• Diabetes

• Target Vessel

1. *Additional Analyses*

• Clinical Predictors of post-PCI FFR ≥0.90

• Clinical Predictors of post-PCI FFR ≤0.80

• Clinical Predictors of post-PCI dPR <0.90

• Clinical Predictors of post-PCI RFR <0.90

• Clinical Predictors of post-PCI CFR <2.0

• Clinical Predictors of Target Vessel Failure at 3 months and 1 year

1. *References*

1. De Bruyne B, Pijls NHJ, Smith L, Wievegg M, Heyndrickx GR. Coronary thermodilution to assess flow reserve - Experimental validation. Circulation. 2001;104(17):2003-6.

2. Pijls NHJ, De Bruyne B, Smith L, Aarnoudse W, Barbato E, Bartunek J, et al. Coronary thermodilution to assess flow reserve - Validation in humans. Circulation. 2002;105(21):2482-6.

3. Fearon WF, Balsam LB, Farouque HMO, Robbins RC, Fitzgerald PJ, Yock PG, et al. Novel index for invasively assessing the coronary microcirculation. Circulation. 2003;107(25):3129-32.

4. Yong AS, Layland J, Fearon WF, Ho M, Shah MG, Daniels D, et al. Calculation of the Index of Microcirculatory Resistance Without Coronary Wedge Pressure Measurement in the Presence of Epicardial Stenosis. Jacc-Cardiovascular Interventions. 2013;6(1):53-8.

5. Garcia-Garcia HM, McFadden EP, Farb A, Mehran R, Stone GW, Spertus J, et al. Standardized End Point Definitions for Coronary Intervention Trials The Academic Research Consortium-2 Consensus Document. Circulation. 2018;137(24):2635-50.

6. Thygesen K, Alpert JS, Jaffe AS, Chaitman BR, Bax JJ, Morrow DA, et al. Fourth universal definition of myocardial infarction (2018). European Heart Journal. 2019;40(3):237-69.
